# Development and Evaluation of Polyvinylpyrrolidone K90 and Poloxamer 407 Self-Assembled Nanomicelles: Enhanced Topical Ocular Delivery of Artemisinin

**DOI:** 10.3390/polym13183038

**Published:** 2021-09-08

**Authors:** Chandrasekar Ponnusamy, Abimanyu Sugumaran, Venkateshwaran Krishnaswami, Rajaguru Palanichamy, Ravichandiran Velayutham, Subramanian Natesan

**Affiliations:** 1 Department of Pharmaceutical Technology, University College of Engineering, Bharathidasan Institute of Technology Campus, Anna University, Tiruchirappalli 620024, Tamil Nadu, India; chandruu0079@gmail.com (C.P.); venkpharm@gmail.com (V.K.); 2 Department of Pharmaceutics, SRM College of Pharmacy, SRM Institute of Science and Technology, Kattankulathur 603203, Tamil Nadu, India; abipharmastar@gmail.com; 3 Department of Life Sciences, School of Life Sciences, Central University of Tamil Nadu, Tiruvarur 627007, Tamil Nadu, India; rajaguru62@gmail.com; 4 Department of Pharmaceutics, National Institute of Pharmaceutical Education and Research (NIPER)—Kolkata, Chunilal Bhawan, 168, Maniktala Main Road, Kolkata 700054, West Bengal, India; directorniperkolkata@gmail.com

**Keywords:** nanomicelles, artemisinin, cornea, toxicity

## Abstract

Age-related macular degeneration is a multifactorial disease affecting the posterior segment of the eye and is characterized by aberrant nascent blood vessels that leak blood and fluid. It ends with vision loss. In the present study, artemisinin which is poorly water-soluble and has potent anti-angiogenic and anti-inflammatory properties was formulated into nanomicelles and characterized for its ocular application and anti-angiogenic activity using a CAM assay. Artemisinin-loaded nanomicelles were prepared by varying the concentrations of PVP k90 and poloxamer 407 at different ratios and showed spherical shape particles in the size range of 41–51 nm. The transparency and cloud point of the developed artemisinin-loaded nanomicelles was found to be 99–94% and 68–70 °C, respectively. The in vitro release of artemisinin from the nanomicelles was found to be 96.0–99.0% within 8 h. The trans-corneal permeation studies exhibited a 1.717–2.169 µg permeation of the artemisinin from nanomicelles through the excised rabbit eye cornea for 2 h. Drug-free nanomicelles did not exhibit noticeable DNA damage and showed an acceptable level of hemolytic potential. Artemisinin-loaded nanomicelles exhibited remarkable anti-angiogenic activity compared to artemisinin suspension. Hence, the formulated artemisinin-loaded nanomicelles might have the potential for the treatment of AMD.

## 1. Introduction

Current treatment options adopted for posterior segment eye diseases such as age-related macular degeneration (AMD) are far from satisfactory due to the limited exposure of therapeutic drugs to the posterior segment of the eye with poor bioavailability [1,2]. Attempts to increase the dose of drugs may lead to posterior segment toxicity [3,4]. Site specific intravitreal injections/implants may improve the localized drug concentration which ultimately results in cataract development, endophthalmitis, haemorrhage and retinal detachment [5]. Topical drops, the most widely adopted method for ocular drug delivery, also suffer from precorneal clearance, lacrimation, tear dilution and tear turnover and result in a low bioavailability [6,7]. Topically administered drugs are penetrated and distributed into the posterior segment of the eye through diffusion across ocular tissues, trans-corneal permeation, direct entry through uvea and lateral diffusion across sclera and conjunctiva [8,9]. The topical application of particulate drug delivery systems such as nanosuspension, nanodispersion, nanocrystals, nanogels and nanomicelles has shown enhanced ocular bioavailability by minimizing the precorneal loss, increasing the corneal residence time, improving the corneal penetration and providing controlled or prolonged drug delivery to the disease site [10].

Nanomicelles are colloidal dispersions of surfactant (s) or polymeric surfactant (s) molecule aggregates with a fairly narrow size distribution of spherically shaped particles (10 to 100 nm). These systems have been reported to improve the solubility of poorly water-soluble drugs by encapsulating them into shells with improved stability [11,12]. Polymeric micelles are generally more stable compared to surfactant micelles and they are mainly used to treat the affected areas due to their bio adhesive properties [13].

Polyvinylpyrrolidone, a synthetic polymer, with the chemical term 1-Ethenyl-2-pyrrolidinone homopolymer, is widely used as a dissolution enhancer for poorly soluble drugs. Due to its strong hydrophilic characteristics, it may improve water penetration and the wettability of the hydrophobic drugs. Poloxamer 407, made up of nonionic polyoxy ethylene–polyoxy propylene copolymers, is used to enhance the solubilization of poorly water-soluble drugs and prolong the drug release [14,15]. The gel to sol transition has been reported for the poloxamers-based ocular delivery of (timolol maleate) upon topical administration [14,16].

Artemisinin is a poorly water-soluble cadinene-type sesquiterpene lactone isolated from Artemisia annua (Family-Compositae), chemically known as (3R, 5aS, 6R, 8aS, 9R, 12S, 12aR)-octahydro-3, 6, 9-trimethyl-3, 12-epoxy-12H-pyrano [4,3-j]-1,2-benzodioxepin-10 (3H)-one. Artemisinin possesses anti-inflammatory and anti-angiogenic activities along with its potent anti-malarial activity. The presence of the endoperoxide bridge (–C–O–O–C–) is responsible for its potent anti-cancer and anti-malarial activities with low toxicity. The endoperoxide bond in artemisinin forms an alkoxyl radical by accepting an electron from heme, thereby artemisinin becomes activated and exerts its anti-angiogenic effect [17,18,19]. Artemisinin has been reported to inhibit both NF-kB activation and the vascular endothelial growth factor (VEGF) which are the main factors in the development of AMD [20,21]. Hence, it is hypothesized that artemisinin can be useful for the treatment of AMD [20,21,22]. Further corneal permeation of artemisinin through the rabbit cornea has not been reported to best of our knowledge.

The aim of the present study was to enhance the aqueous solubility of artemisinin by using the combination of PVP K90 and poloxamer 407 nanomicelles and to evaluate its corneal permeability. Furthermore, the hemolytic potential, genotoxicity and critical micellar concentration of the developed nanomicelles were also evaluated. The anti-angiogenic potential of the developed nanomicelles was evaluated by chick embryo chorioallantoic membrane (CAM) assay.

## 2. Materials and Methods

### 2.1. Materials

Artemisinin was obtained from Herbochem, (Hyderabad, India). Polyvinylpyrrolidone (PVP K90) (Molecular weight—40,000 Da) and Poloxamer 407 (Molecular weight—95,000–110,000 Da) were procured from BASF Corporation, Mumbai, India. Potassium dihydrogen orthophosphate and sodium hydroxide were purchased from SD fine chemicals, Mumbai, India. All other solvents and reagents used were of HPLC grade. Milli Q water was used for HPLC analysis.

### 2.2. Compatibility Study

The drug and excipient compatibility were assessed by the Fourier Transform Infra-Red spectrometer (Spectrum Two, Perkinelmer, Waltham, MA, USA). The potassium bromide (KBr) pellets were prepared by grinding KBr with respective samples (artemisinin/PVP K90/poloxamer 407/physical mixture of artemisinin with excipients, processed mixture and/artemisinin loaded nanomicelles) and pressed into transparent pellets using a hydraulic press [22]. The IR spectra were recorded at the region of 4000–400 cm^−1^.

### 2.3. Preliminary Screening for Nanomicelles Formation Capacity

Different mass ratios of PVP K90 and poloxamer 407 (1:1, 1:2, 1:3, 1:4, 1:5, 2:1, 2:2 etc., [1 = 5%]) and 100 mg of artemisinin were dissolved in ethanol, stirred to form homogeneous ethanolic solution and dried under vacuum to form artemisinin-loaded polymeric particles. The obtained mass was treated with sterile water, vortexed to form stable nanomicelles and examined for turbidity after 24 h.

### 2.4. Design of Experiment (DoE)

The central composite rotatable design–response surface methodology (CCRD–RSM) at two factor and three levels (i.e., −1, 0, +1) was used to optimize the artemisinin-loaded nanomicelles. The polymer (PVP K90) and polymeric surfactant (Poloxamer 407) concentrations were kept as input variables. The particle size and transparency of the developed formulations were kept as the response variables. The concentrations of the PVP K 90 (0–10% *w/v*) and poloxamer 407 (0–5% *w/v*) were selected based on the acceptable levels reported by USFDA for ophthalmic use [16,17]. The experimental design and statistical analysis of the obtained data were performed using the Design Expert Software (Version 6, Stat-Ease Inc., Minneapolis, MN, USA).

### 2.5. Preparation of Artemisinin Loaded Nanomicelles

A weighed amount of artemisinin was dissolved along with PVP K90 and Poloxamer 407 (2:1) in ethanol and was spray dried to form artemisinin-loaded polymeric particles. These polymeric particles were dissolved in a phosphate buffer with a pH of 7.4 and were vortexed to form stable artemisinin-loaded nanomicelles (Table 1).

### 2.6. Determination of the Critical Micellar Concentration (CMC)

The critical micellar concentration (CMC) of artemisinin-loaded nanomicelles was determined by the pyrene-based fluorescent probe method clearly described by Li et al. and Mohr et al. [23,24]. In this method, a solution of 10 mM pyrene was prepared using a phosphate buffer saline (PBS). Artemisinin nanomicelles and blank nanomicelles were prepared at a concentration of 30–1500 µg/mL using a saline phosphate buffer. Pyrene was added in order to achieve a 0.1 μM solution in concentration and was incubated for 30 min at room temperature in the dark. The sample was excited at 336 nm and fluorescence intensity was measured with dual emission at 375/384 nm using a microplate reader (Molecular Devices, San Jose, CA, USA). The ratio of intensity (I384/I375) was computed and compared to the nanomicelles’ log concentrations.

### 2.7. Characterization of Artemisinin-Loaded Nanomicelles

#### 2.7.1. Transparency

The transparency of the formulated artemisinin-loaded nanomicelles was evaluated at 400 nm by a UV spectrophotometer using distilled water as a blank [25].

#### 2.7.2. Cloud Point

The cloud point of artemisinin-loaded nanomicelles was examined by placing a 100-fold diluted sample with water in a water bath and subjecting it to increases in temperature gradually. The temperature at which a decrease in transmittance occurred due to cloudiness formation was noted visually as the cloud point [26].

#### 2.7.3. Particle Size and Zeta Potential

The average particle size and zeta potential of the developed artemisinin-loaded nanomicelles was determined by using a Zetasizer (Nano ZS, Malvern Instruments, Malvern, UK). The homogeneity of the particle size distribution was indicated by its polydispersity index (PDI).

#### 2.7.4. Transmission Electron Microscopy (TEM)

Transmission electron microscopy (Philips EM-430; Philips Electronics, Eindhoven, The Netherlands) was employed to investigate the surface morphology of nanomicelles and it was operated at a driving voltage of 200 kV. A drop of 1.3% phosphotungstic acid was applied to the samples before they were placed over the carbon-coated copper grid. The grid was vacuum dried before being mounted on a grid holder and its morphology was examined.

#### 2.7.5. Drug Content

The amount of artemisinin present in the artemisinin-loaded nanomicelles was checked by RP-HPLC (Shimadzu Corporation, Kyoto, Japan, LC- 20AD). Data acquisition was performed using spinchrome-1 software. A reverse phase phenomenex-C18 (5 μm, 4.6 mm × 250 mm) analytical column was used. The mobile phase consisting of the mixture of acetonitrile: water (65:35% *v/v*) was used at a flow rate of 1.0 mL/min and the UV detection was carried out at 219 nm [27].

### 2.8. In Vitro Hemolytic Potential

The in vitro hemolytic potential of the nanomicelles was evaluated by the method described by Amin et al. (2006) [28]. Briefly, 100 µL of blank nanomicelles were combined with 900 μL of fresh peripheral blood from healthy human volunteers and equilibrated at 25 °C for 2 min and centrifuged at 1900× *g* at 25 °C for 5 min. The precipitates were washed 4 times with 5 mL of normal saline after discarding the supernatant. The unlysed red blood cells were treated and vortexed with 4 mL of sterile water for supplementation with haemoglobin release and had a centrifugal effect for 5 min at 1900× *g*. The obtained supernatant was diluted with distilled water and absorbance was measured at 540 nm. Normal saline (0% lysis) and a 1% sodium carbonate solution (100% lysis) were used as negative and positive controls, respectively. The hemolytic capacity of the nanomicelles was estimated by the formula:

(1) $$\%\;Hemolysis=\frac{Negative\;Control-Blank\;Nanomicelles}{Negative\;Control-Positive\;Control}\times 100$$

### 2.9. Evaluation of the Genotoxicity of the Nanomicelles (Alkaline Comet Assay)

The toxic effect of the blank nanomicelles (BNM 2 and BNM 3) on the genetic materials of the cells was evaluated by the alkaline comet assay as previously described by Natesan et al. [29] using cells treated with H_2_O_2_ (100 µM) as the positive control.

### 2.10. In Vitro Drug Release

The in vitro drug release of the artemisinin-loaded nanomicelles was evaluated using a dialysis bag method (cut off 5000 Da, Himedia, Mumbai, India). The drug-loaded nanomicelles (1.0 mL) were placed in a dialysis bag which was hermetically sealed and suspended in 50 mL of a phosphate buffer solution at a pH of 7.4. The temperature was maintained at 34 ± 0.1 °C using a closed double jacketed thermostatic chamber and stirred at 600 RPM using a magnetic stirrer [30,31]. Aliquots (2.0 mL) were withdrawn at predetermined intervals and replaced by an equal volume of the fresh dialyzing medium. The samples were analyzed using RP-HPLC.

### 2.11. In Vitro Trans-Corneal Permeation Studies

The in vitro trans-corneal permeation effect of artemisinin-enriched nanomicelles were studied with a modified side-by-side of a Franz diffusion cell. Both chambers (donor and receptor) were built with an internal water jacket for water circulation and side braces to keep the system temperature constant. The protocol for animal research was authorized by the Institutional Animal Ethical Committee and animals were kept in accordance with approved guidance. In combination with the 2–4 mm surrounding scleral tissue, the male albino rabbit cornea was carefully removed and rinsed with cold saline. The excised cornea was positioned between the cells of the donor and the receivers in such a manner to allow the cornea’s epidermal surface to face the donor compartment with a diffusion area of 0.78 cm^2^. The phosphate buffer with a pH of 7.4 was deposited into the compartment and maintained at 34 ± 0.1 °C followed by a magnetic stirring of 150 rpm. The pre-heated (34 °C) drug-loaded nanomicelles were inserted into the donor cell at specified time intervals and the samples of the reservoir cell (0.3 mL) were removed and replaced by fresh media. The samples were evaluated using the RP-HPLC technique as previously described by Chandrasekar et al. [27].

### 2.12. Apparent Permeability Coefficient

The apparent permeability coefficient was calculated using the following formula:

(2) $$Papp=\frac{\Delta Q}{\Delta t}.\frac{1}{\left(A.Co.60\right)}$$

where, Δ*Q*/Δ*t* is the flux across the cornea (ng/min), *A* is the diffusion area exposed, *Co* is the starting concentration of the artemisinin in a donor region, and 60 is regarded as the minute-to-second factor. The corneal flux was derived from the path of the regression line, which was drawn for the amount of drug (*Q*) penetrated by time (*t*) [32,33].

### 2.13. Histological Examination of the Drug-Permeated Cornea

The corneal permeate tissue obtained after the permeation study was washed with a phosphate buffer with a pH of 7.4 and was stored in a 10% formalin solution. The corneal permeate tissue was dehydrated with ethanol, embedded in paraffin, cut into vertical sections using microtome, stained with hematoxylin eosin and observed under a light microscope for any pathological change using untreated corneal tissue as a control [34].

### 2.14. Evaluation of the Anti-Angiogenic Effect of Artemisinin Nanomicelles Using a Chorioallantoic Membrane Assay (CAM Assay)

The anti-angiogenic effect of the nanomicelles formulation was evaluated using a Chorioallantoic Membrane Assay (CAM) as described by Ponnusamy et al. and Velpandian et al. [22,35]. Fertilized chicken eggs were incubated in an incubator at 37 °C for 3 days. The eggs were turned horizontally many times and then swabbed with 70% alcohol on the third day. By withdrawing the albumin (2.0 mL) from the fertilized eggs, the growing CAM was separated from the eggshell and an incision was made to create a window which was utilized as an entry point to the CAM. Sterile parafilm was used to close the window. The viable eggs were horizontally inserted and incubated for up to 5 days. On the 5th day the embryos were imaged by digital camera to reveal excising blood vessels on the window. A solution of artemisinin/blank nanomicelles/artemisinin-loaded nanomicelles on an impregnated filter paper disc (each 50 μg/disk) was put directly onto an exposed blood vessel using sterile surgical forceps on an increasing CAM and further incubated for 2 days. On day 7, the filter paper discs were carefully removed from the CAM and their anti-angiogenic impact was assessed and photographed at the sample applied area. A semi-quantitative score system was used to access the anti-angiogenic impact.

### 2.15. Stability Studies

The stability study of the artemisinin-loaded nanomicelles was carried out based on ICH guidelines by storing the samples at 40 ± 2 °C/75 ± 5% RH for 180 days in the stability chamber.

### 2.16. Statistical Analysis

Triplication of all experiments were completed and the data are given as a mean ± standard deviation. The student’s “*t*” test was used to examine statistical data.

## 3. Results and Discussions

### 3.1. Compatibility Studies (FTIR)

The interactions between the drug and the excipients were analyzed by comparing the FTIR spectra of the pure drug, the individual excipients, the physical mixture, the processed mixture of the polymer and the polymeric surfactant and the artemisinin-loaded nanomicelles.

**Figure 1:**
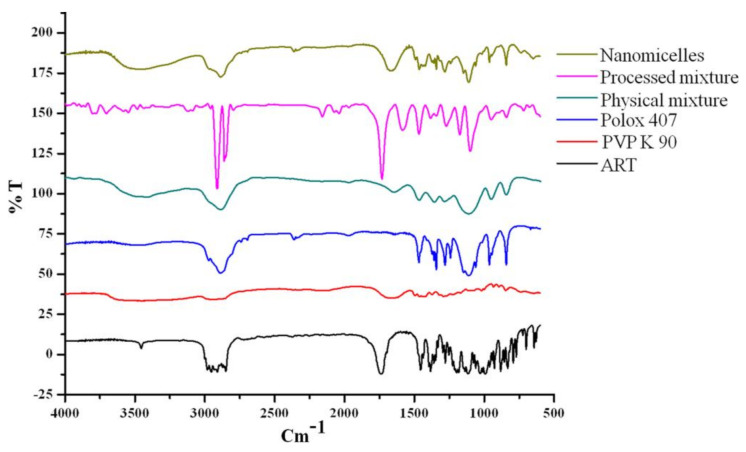
FTIR spectrum of artemisinin, PVP K 90, poloxamer 407, physical mixture, processed mixture and artemisinin nanomicelles.

An artemisinin IR spectrum contains unique bands of IR regions such as, 1116 cm^−1^ for the C–O–O–C bending vibrations of the endoperoxide ring, 1736 cm^−1^ for the C=O lactone ring stretching and 1012 cm^−1^, 1200 cm^−1^ and 3455 cm^–1^ stretching for the C–O, C–O–C and O–H stretching vibrations, respectively (Figure 1). The symmetric CH_3_ stretch was marked by identifying the fermi resonance at 2952 cm^–1^ with overtones of methyl bending. The IR absorption bands at 883 cm^–1^ and 831 cm^–1^ were responsible for O–O–C and O–O stretching as the boat/twist form of 1, 2, 4-trioxane [36]. The characteristic IR absorption pattern of PVP K90 was shown in the bands of 3447 cm^−1^ with O–H stretching vibrations, C–H stretching vibration at 2886 cm^−1^, C=O carbonyl stretching at 1655 cm^−1^, C–H stretching at 1375 cm^−1^, and C–N stretching vibration at 1281 cm^−1^ [18]. The properties of Poloxamer 407 include the absorption bands for aliphatic IR vibration C–H at 2921 cm^−1^, the vibration C–O stretches at 1117 cm^−1^ and the vibration O–H stretches at 3452 cm^−1^ [37,38].

The IR spectra of artemisinin nanomicelles and the processed mixture shows the presence of C=O stretching at 1644 and 1653 cm^−1^ (carbonyl bond) and 1117 cm^−1^ for C–O (ether bands) stretching vibrations which confirms the interaction between the poloxamer and PVP; this interaction was absent in the physical mixture [39]. The artemisinin-loaded nanomicelles exhibited a shift at the C=O stretching vibrations from 1736 cm^−1^ to 1644 cm^−1^ and 1653 cm^−1^, respectively. The changes in the carbonyl (C=O) group of the lactone ring in artemisinin might contribute towards the artemisinin solubility.

The formation of a weaker hydrogen bond between the lactone ring carbonyl group (C=O) and the poloxamer hydroxy group could contribute to the enhancement of artemisinin solubility [40]. Moreover, the muster between the poloxamer hydroxy group and the PVP ketone group might increase the hydrophobic micelle corona, which might increase artemisinin solubility.

### 3.2. Preliminary Screening for Nanomicelles Formation Capacity

The stable nanomicelles were screened based on the transparency and the formation of a precipitate upon storage for up to 24 h. Poloxamar 407 and PVP k90 (1:2) produced clear nanomicelles, whereas other lower ratios produced unclear nanomicelles. The higher ratio of poloxamer 407 to PVP K 90 produced visually clear nanomicelles, but the amount required for the formation of nanomicelles was higher than the amount permitted to be used for ocular application as per USFDA [41,42]. The preliminary trial experiments indicated that the polymer and the polymeric surfactant concentration affects the transparency and particle size of the nanomicelles.

### 3.3. Experimental Design

**Figure 2:**
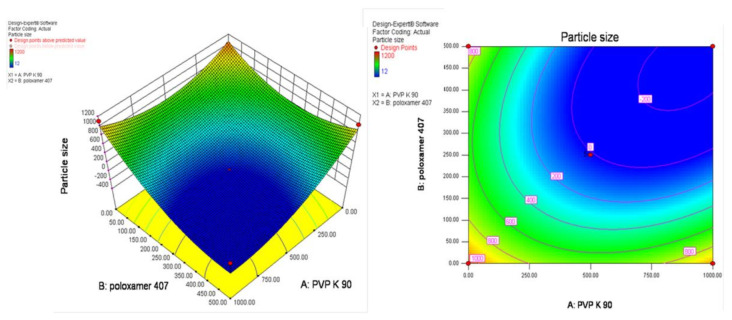
Three-dimensional (3D) and Contour plots of the response surface method graph for the effect of PVP K 90 and poloxamer 407 concentrations on the particle size of nanomicelles.

**Figure 3:**
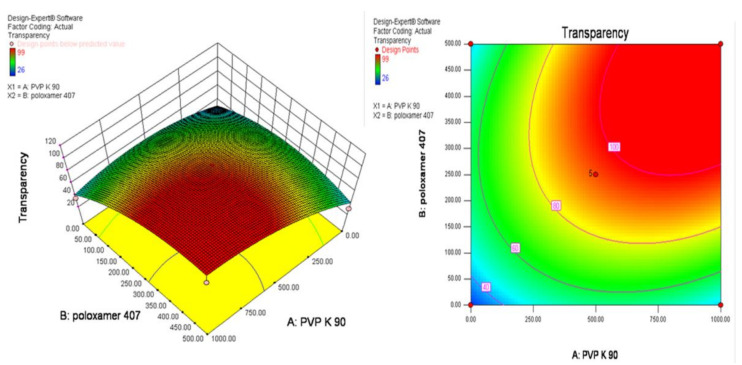
Three-dimensional (3D) and Contour plots of the response surface method graph for the effect of PVP K 90 and poloxamer 407 concentrations on the transparency of nanomicelles.

The optimization to screen the smaller particle size with transparent artemisinin-loaded nanomicelles was obtained using central composite rotatable design–response surface methodology (CCRD–RSM). The optimization layout is shown in Table 2, and three-dimensional (3D) RSM graphs and contour graphs are shown in Figure 2 and Figure 3, respectively. The mathematical relationships constructed for the studied response variables of particle size and transparency are expressed as given below in Equations (2) and (3). The best fitting was obtained with the quadratic model. The R-Squared, adjusted R-Squared, predicted R-Squared and adequate precision of the particle size were 0.5818, 0.2830, −1.9741 and 3.269, respectively. Similarly, the R-Squared, adjusted R-Squared, predicted R-Squared and adequate precision of the transparency of the systems were 0.7968, 0.6517, −0.4449 and 5.865, respectively.

Final Equation in Terms of Coded Factors:

Particle size = + 44.31 − 4.55 × A − 2.50 × B − 23.00 × A × B+ 36.35 × A^2^ + 20.85 × B^2^ (3)

Transparency = + 96.00 + 0.26 × A + 0.97 × B + 26.25 × A × B − 22.31 × A2 − 19.31 × B2(4)

where A = Concentration of poloxamer, B = Concentration of PVP K 90.

The increasing concentration of poloxamer 407 contributed negatively towards the particle size and positively towards the transparency of nanomicelles, whereas PVP K90 exhibited opposite results. RSM diagrams showed that the higher the concentration of PVP K90 and poloxamer 407, the higher the particle size produced with a lower transparency. Transparent nanomicelles and lower particle size nanomicelles were obtained effectively within the limit of 5 to 10% of PVP K 90 and 2.5 to 5% of poloxamer 407.

### 3.4. Preparation of Artemisinin-Loaded Nanomicelles

Three different concentrations of PVP K90 and poloxamer 407 are permitted for ophthalmic applications at a 2:1 ratio and were used to formulate the artemisinin-loaded nanomicelles [30]. Previously, it has been reported that the addition of poloxamer in celecoxib microspheres showed a 5-fold enhanced solubility and dissolution rate (98.0% release in 30 min) of celecoxib. In addition, the viscosity of ocular formulations became enhanced upon the incorporation of the poloxamer and PVP K90 as thickening agents and this may retain the incorporated drugs for a prolonged period of time [43].

### 3.5. Determination of Critical Micellar Concentration

Poloxamers are non-ionic polymeric surfactants and PVP is a non-ionic homo copolymer. These two non-ionic polymers are grafted together with an increased solubility. The artemisinin carbonyl group may combine with the poloxamer hydroxyl group to produce self-assembled micelles in water [39].

**Figure 4:**
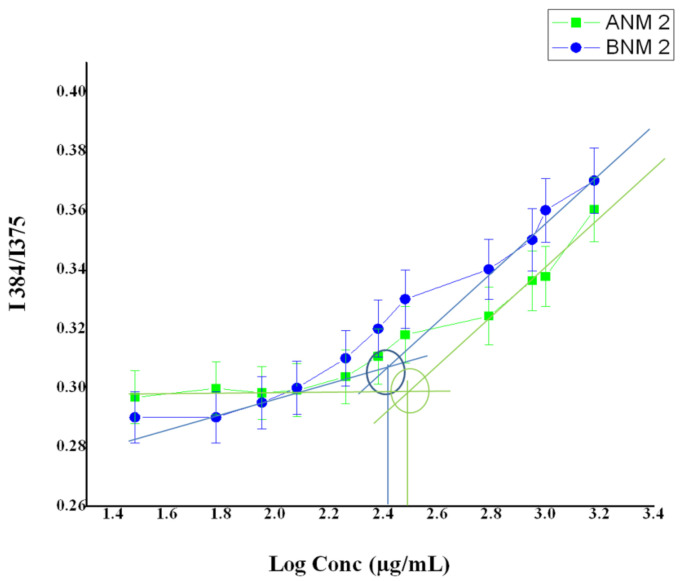
CMC of the blank nanomicelles and artemisinin nanomicelles by a pyrene-based fluorescent probe method.

The CMC results show that the addition of pyrene to the aqueous dispersion of blank nanomicelles and artemisinin-loaded nanomicelles causes a substantial quenching of pyrene fluorescence (Figure 4). An increase in the concentration of PVP K 90 and poloxamer 407 causes a red shift in the emission and the intensity ratio I384/I375 of pyrene is gradually increased. The CMC observed for the blank nanomicelles and artemisinin-loaded nanomicelles was around 263 ± 3 µg/mL and 295 ± 3 µg/mL, respectively.

### 3.6. Characterization of Artemisinin-Loaded Nanomicelles

#### 3.6.1. Transparency

The artemisinin nanomicelles that were formed were clear and transparent in nature. The transparency of the formulations decreased from 99.0% to 94.0% upon the increase in the concentration of PVP K 90 and poloxamer 407 (Table 3).

#### 3.6.2. Cloud Point Measurement

The cloud point of artemisinin-loaded nanomicelles was around 68–70 °C (Table 3). The cloudiness that appeared in the tested artemisinin-loaded nanomicelle disappeared upon the decrease in temperature just below the cloud point and this happened within a few minutes. These results indicate that the developed artemisinin-loaded nanomicelles might be stable at body temperature. The micellar enlargement and packing were formed due to the dehydration of the polymer block upon the increase in temperature. The non-ionic polymeric surfactant poloxamer undergoes phase separation at high temperatures due to the dehydration of the polyethylene oxide moiety [26].

#### 3.6.3. Particle Size and Zeta Potential

The mean particle size and charge of the artemisinin-loaded nanomicelles was found to be 41 to 51 nm and –5 mV to –12 mV, respectively. The polydispersity index ranges from 0.353 to 0.422 (Table 3). The particle size of artemisinin-loaded nanomicelles increased upon the increase in the concentrations of the polymer and the polymeric surfactant [16,44].

#### 3.6.4. Morphology and Drug Content

**Figure 5:**
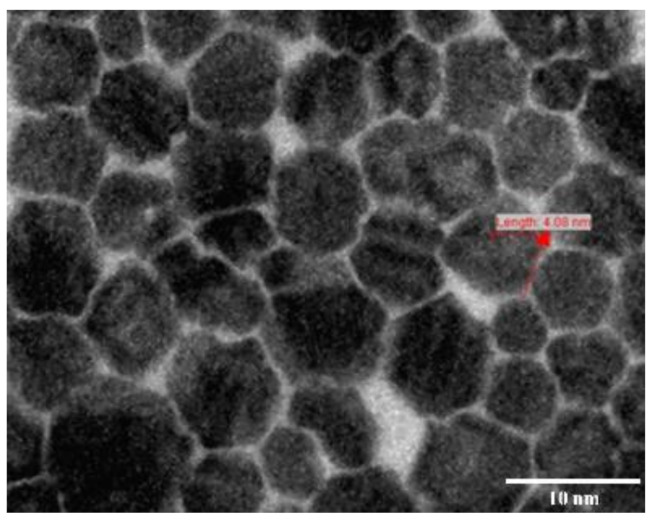
Transmission electron photomicrograph of artemisinin nanomicelles (ANM 2).

The TEM photomicrograph (Figure 5) shows that the dispersed particles had a smooth surface with a spherical shape and were distributed uniformly. The fragmentation pattern shows the hydration of the polymer blocks upon solubilizing in PBS. The surface of the dispersed particle photomicrograph displays the entrapped drug particles and the drug particle distribution within the nanomicelles. The amount of artemisinin present in the artemisinin-loaded nanomicelles ranged from 98 to 99% *w/v*.

### 3.7. In Vitro Hemolytic Potential

**Figure 6:**
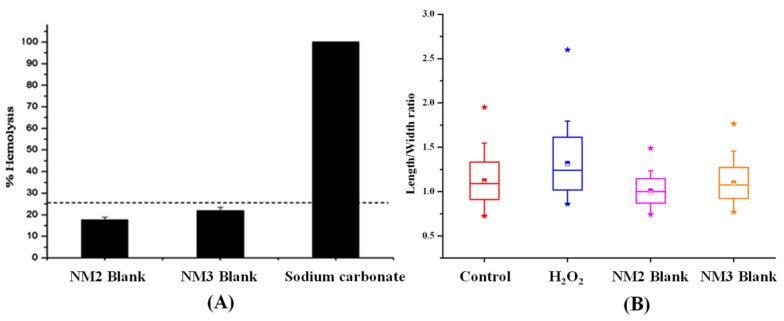
(**A**) In vitro hemolytic potential of blank nanomicelles in human peripheral blood. (**B**) In vitro genotoxicity of blank nanomicelles in human peripheral blood lymphocytes by an alkaline comet assay. * The minimum and maximum value (outliers) of the data.

The non-irritant potential of the blank nanomicelles with a concentration of PVP K90 and poloxamer 407 (8:4 and 10:5%) as evaluated by hemolytic studies is shown in Figure 6A. Topical ocular drops are intended for delivering the drugs through the cornea, aqueous humor and the vitreous humor to the retina. In wet AMD, a leakage of blood and fluid occurs in the macula region of the retina. The vehicle with the hemolytic properties will destroy the leaked blood cells that cause an enlargement of the macula. The blank nanomicelles BNM 2 and BNM 3 showed 20% and 22.5% hemolysis, respectively. It was observed that the percentage of hemolysis of the red blood cells produced by blank nanomicelles was within the acceptable range (25%). Hence, the formulated nanomicelles might be safe for topical administration.

### 3.8. Evaluation of the Genotoxicity of the Nanomicelles by the Alkaline Comet Assay

**Figure 7:**
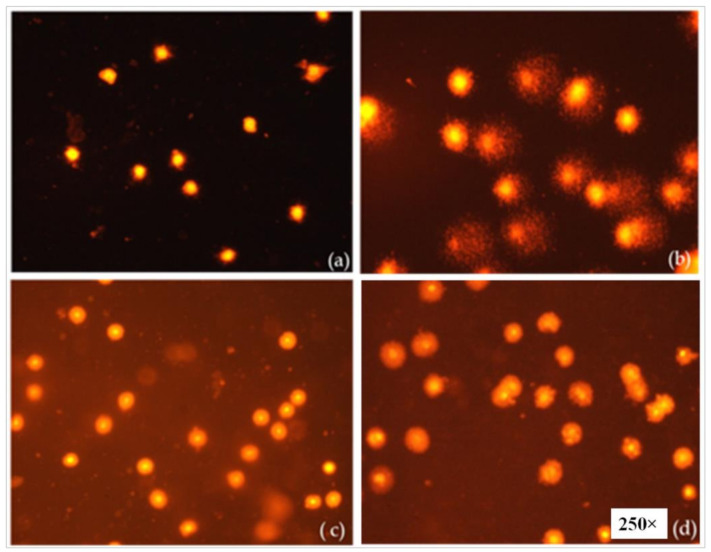
Photomicrographs of Ethidium/Bromide-stained white blood cells under fluorescent microscope. Control cells (**a**), hydrogen peroxide treated cells (**b**), blank NM 2 treated cells (**c**), blank NM 3 treated cells (**d**).

The genotoxic effect of the artemisinin-loaded nanomicelles was evaluated by an alkaline comet assay and the results are displayed in Figure 6B. The high concentration of the polymer (10%) and the polymeric surfactant (5%) containing blank nanomicelles (NM 3) produced no significant DNA damage (Figure 7) in comparison with hydrogen peroxide (positive control). The formulated artemisinin-loaded nanomicelles did not produce any remarkable damage in mammalian cells also. Hence, the developed artemisinin-loaded nanomicelles with higher concentrations of the polymer and the polymeric surfactant might be useful for ocular topical drug delivery.

### 3.9. In Vitro Drug Release

**Figure 8:**
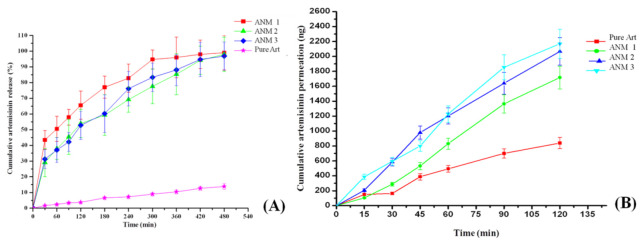
(**A**) In vitro drug release of artemisinin from artemisinin nanomicelles and pure drug suspension. (**B**) In vitro corneal permeation of artemisinin from artemisinin nanomicelles and pure drug suspension.

The in vitro drug release studies indicate that around 96.0 to 99.0% of artemisinin was released from artemisinin nanomicelles ANM 1, ANM 2, ANM 3 and only 14% of artemisinin was released from pure drug suspension within 8 h (Figure 8A,B). The artemisinin-loaded nanomicelles (ANM 1 and ANM 2) showed a higher drug release than the nanomicelles formulated with higher concentrations of 10% PVP K 90 and 5% poloxamer 407 (ANM 3) and drug suspensions. In the present study, artemisinin release from the nanomicelles was indirectly proportional to the polymer and polymeric surfactant concentrations of the formulation. This might be due to the encapsulation of the drug particles inside the nano structure of the formulation. The slowest release of artemisinin suspensions was observed due to its hydrophobicity which leads to a floating of the drug powder in the dissolution medium. The increased dissolution rate of artemisinin-loaded nanomicelles might be due to the reduction in drug crystal size, the carrier solubilization effect, the reduction in drug aggregation and agglomeration, the conversion of the amorphous state and increased drug wettability [45].

A similar result was reported for the in vitro release of timolol maleate from the ocular gel, where the release increased with a decreased concentration of pluronic due to the changes in the structural configuration of the polymeric gel. El-Kamel [43] emphasized that the increased concentration of Pluronic127 leads to a decrease in the amount of the drug released. It might be due to the reduction in the number and dimension of water channels through the polymeric gel.

### 3.10. In Vitro Trans-Corneal Permeation of All the Nanomicelles

The in vitro corneal permeation of artemisinin from the artemisinin-loaded nanomicelles was evaluated through the excised rabbit cornea. The permeation of artemisinin through the excised cornea followed the order of ANM 3 > ANM 2 > ANM 1 > drug dispersion (Figure 8B). The maximum amount of artemisinin (2169 ng) permeated from ANM 3 which is higher than that of drug suspension by 2.5-fold. Similarly, 2067 ng and 1717 ng of artemisinin permeated from ANM 2 and ANM 1 and 839 ng of artemisinin permeated from the artemisinin drug suspension. The permeation of artemisinin increased gradually upon the increased concentration of PVP and poloxamer 407. It has been reported that the ocular bioavailability of drugs is enhanced with an increased concentration of surfactants [33,46]. The ocular bioavailability of timolol maleate from ocular gel increased (2 to 2.5-folds) with an increased concentration of pluronics (15–25%). The increased corneal permeation observed with artemisinin-loaded nanomicelles might be due to the presence of poloxamer 407 which increases the solubility of the artemisinin, removes the phospholipids from the epithelial cell membrane without damage and relaxes the epithelium cell junction which leads to the influx of hydrophilic compounds through the cornea [33,47].

### 3.11. Apparent Permeability Coefficient (Papp)

**Figure 9:**
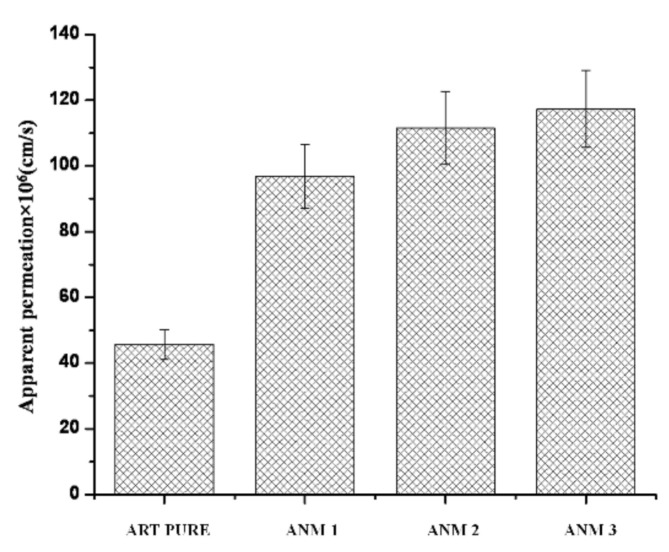
Apparent permeability coefficient of artemisinin from pure drug suspension and artemisinin nanomicelles.

The papp was calculated from the corneal permeation profiles of nanomicelles and the data are shown in Figure 9. The Papp of artemisinin from artemisinin-loaded nanomicelles ANM 3, ANM 2, ANM 1 and pure drug suspension was found to be 117.31 × 10^6^ cm/s, 111.55 × 10^6^ cm/s, 96.84 × 10^6^ cm/s and 45.63 × 10^6^ cm/s, respectively. The enhancement of Papp was 2.57, 2.44 and 2.12-fold for ANM3, ANM2 and ANM1 artemisinin-loaded nanomicelles when compared with artemisinin suspension. The corneal permeability of artemisinin significantly increased with an increased concentration of the polymeric surfactant [46]. It has been reported that the passive corneal transport of the drugs is improved in the presence of a surfactant [47]. It was observed that the apparent permeation coefficient of the artemisinin-loaded nanomicelles was directly proportional to the concentration of the surfactants.

### 3.12. Histological Examination of the Drug-Permeated Cornea

**Figure 10:**
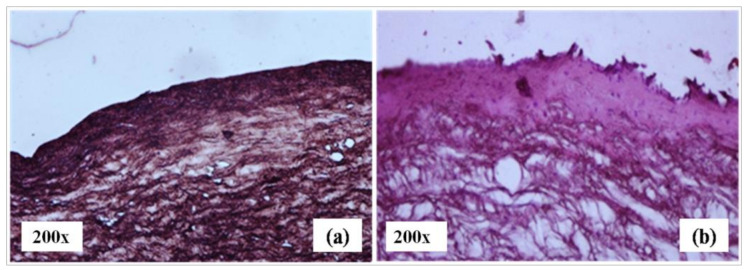
Histopathological image of a normal cornea (**a**) and an ANM 2 penetrated cornea (**b**).

The realistic mechanism of drug permeation through the cornea was examined by histological evaluation (Figure 10). The photograph of the cornea after 2 h of corneal permeation shows that the surface of the epithelium and stroma were modified in comparison to the control. The pore sizes of the cornea and stroma expanded; hence, the drug permeation through cornea may be enhanced without damaging the membrane [48].

The formulations consisting of Poloxamer 407 and PVP may become trapped in the corneal epithelium’s lipid bilayer which may affect the physical characteristics of the epithelial membrane and cause membrane or corneal solubilization of artemisinin. The use of the formulation containing these combined surfactants also dissolves the phospholipids of epithelial cell membranes and establishes a trans-corneal pathway, allowing the drug to be entrapped in these areas more easily [48].

The use of the surfactant in ocular tissues was reported to show reversible changes in the histology and improve the penetration of encapsulated drugs; thereby, frequent administration and side-effects may be minimized [49].

### 3.13. Anti-Angiogenic Effect of Artemisinin-Loaded Nanomicelles

**Figure 11:**
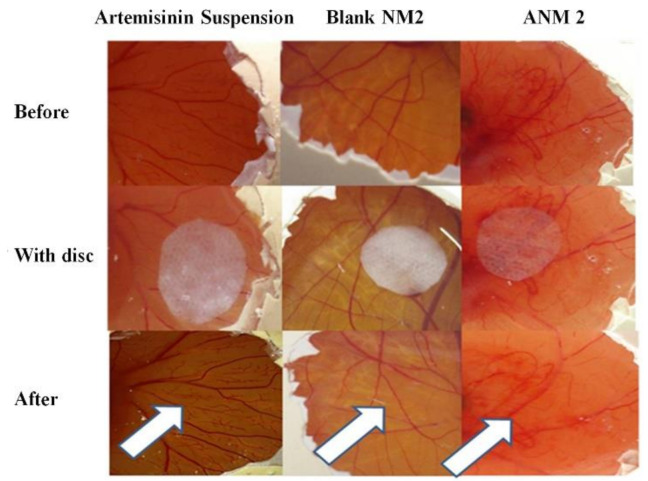
Anti-angiogenic effect of artemisinin drug suspension, drug free NM 2 and artemisinin nanomicelles (ANM 2).

The artemisinin-loaded nanomicelles-treated embryo below the filter paper disc showed changes in blood vessels such as a reduced branching pattern and a capillary free area after 24 h of treatment (Figure 11). However, these changes were not noticeable in blank nanomicelles. The artemisinin suspension-treated embryo also showed very minor changes in the branching patterns of the blood vessels compared to artemisinin nanomicelles-treated CAM.

The small capillaries below the filter paper disc in the developing CAM were absent, but the larger pre-existing vessels remain unaffected. The improved solubility of artemisinin in artemisinin-loaded nanomicelles might be responsible for the qualitative improvement in the anti-angiogenic effect. The anti-angiogenic agent was used to stop and eradicate the existing and newly formed blood vessels. Hence, the developed artemisinin-loaded nanomicelles are expected to have the potential to treat the wet AMD.

Blank nanomicelles did not produce any remarkable changes in the branching pattern of the blood vessels. It was also observed that small capillaries and larger preexisting vessels were unaffected below the disc. This suggests that the surfactant concentration used in the formulation does not produce any irritation and discoloration of the chorioallantoic membrane. Hence, the formulated artemisinin-loaded nanomicelles can be used for ophthalmic drug delivery.

### 3.14. Stability Studies

Artemisinin-loaded nanomicelles were examined at the end of every month to assess their instability parameters such as particle size, turbidity and drug content. The particle size and drug content of the formulations showed no significant changes during the study period.

## 4. Conclusions

Topical formulations of artemisinin-loaded nanomicelles were developed using PVP K90 and poloxamer 407 in the ratio of 2:1. An increase in the concentration of the polymers showed an enhanced particle size of the nanomicelles. The nanomicelles made with 8% of PVP K90 and 4% of poloxamer 407 (ANM 2) showed a moderate drug release and a higher corneal permeation of artemisinin when compared with artemisinin suspension. The artemisinin-loaded nanomicelles also showed a better anti-angiogenic potential than artemisinin suspension. Hence, developed artemisinin nanomicelles using 8% of PVP K 90 and 4% of Poloxamer 407 may have the potential for an effective treatment of AMD.

## Figures and Tables

**Table 1:** Composition of artemisinin nanomicelles (ANM) and blank nanomicelles (BNM).

Nanomicelles	Composition (%)		
	Polyvinyl Pyrolidone K90	Poloxamer 407	Artemisinin
ANM 1	5	2.5	0.05
ANM 2	8	4	0.05
ANM 3	10	5	0.05
BNM 1	5	2.5	-
BNM 2	8	4	-
BNM 3	10	5	-

**Table 2:** Experimental design variables and the observed responses in the response surface methodology for the formulation of nanomicelles.

Run	Factor 1 A: Poloxamer 407	Factor 2 B: PVP K 90	Response 1 Particle Size nm (PDI)	Response 2 Transparency %
1	0.00	0.00	44 (0.36)	96
2	−1.00	1.00	106 (0.54)	44
3	1.00	1.00	51 (0.42)	94
4	0.00	0.00	44 (0.36)	96
5	0.00	0.00	44 (0.36)	96
6	0.00	1.41	98 (0.43)	46
7	−1.00	−1.00	41 (0.35)	99
8	1.00	−1.00	78 (0.33)	44
9	−1.41	0.00	156 (0.41)	33
10	1.41	0.00	143 (0.56)	38
11	0.00	−1.41	139 (0.86)	37
12	0.00	0.00	44 (0.36)	96
13	0.00	0.00	44 (0.36)	96

**Table 3:** Evaluation of particle size, PDI, zeta potential, transparency and cloud point of the nanomicelles.

Formulation Code	Particle Size (nm)	PDI	Zeta Potential (mV)	Transparency (%)	Cloud Point (°C)
BNM 1	32 ± 0.7	0.31 ± 0.13	−4.0 ± 1.6	99 ± 1.1	69 ± 1
BNM 2	34 ± 0.3	0.30 ± 0.11	−7.0 ± 1.1	97 ± 1.4	68 ± 1
BNM 3	39 ± 0.6	0.34 ± 0.21	−10.0 ± 1.3	95 ± 1.8	69 ± 2
ANM 1	41 ±0.9	0.35 ± 0.11	−5.0 ± 1.2	99 ± 1.3	70 ± 1
ANM 2	44 ± 1.1	0.36 ± 0.12	−9.2 ± 1.4	96 ± 1.9	69 ± 2
ANM 3	51 ± 2.1	0.42± 0.13	−12.0 ± 2.8	94 ± 2.1	68 ± 1

Data are mean ± SD, n = 3.

## References

[1] Stahl A. (2020). The Diagnosis and Treatment of Age-Related Macular Degeneration. Dtsch. Arztebl. Int..

[2] Nayak K., Misra M. (2018). A review on recent drug delivery systems for posterior segment of eye. Biomed. Pharmacother..

[3] Rodrigues G.A., Lutz D., Shen J., Yuan X., Shen H., Cunningham J., Rivers H.M. (2018). Topical Drug Delivery to the Posterior Segment of the Eye: Addressing the Challenge of Preclinical to Clinical Translation. Pharm. Res..

[4] Gokulgandhi M.R., Vadlapudi A.D., Mitra A.K. (2012). Ocular toxicity from systemically administered xenobiotics. Expert Opin. Drug Metab. Toxicol..

[5] Varela-Fernández R., Díaz-Tomé V., Luaces-Rodríguez A., Conde-Penedo A., García-Otero X., Luzardo-Álvarez A., Fernández-Ferreiro A., Otero-Espinar F.J. (2020). Drug Delivery to the Posterior Segment of the Eye: Biopharmaceutic and Pharmacokinetic Considerations. Pharmaceutics.

[6] Agrahari V., Mandal A., Agrahari V., Trinh H.M., Joseph M., Ray A., Hadji H., Mitra R., Pal D., Mitra A.K. (2016). A comprehensive insight on ocular pharmacokinetics. Drug Deliv. Transl. Res..

[7] Sapino S., Chirio D., Peira E., Abellán Rubio E., Brunella V., Jadhav S.A., Chindamo G., Gallarate M. (2019). Ocular Drug Delivery: A Special Focus on the Thermosensitive Approach. Nanomaterials.

[8] Gote V., Sikder S., Sicotte J., Pal D. (2019). Ocular Drug Delivery: Present Innovations and Future Challenges. J. Pharmacol. Exp. Ther..

[9] Swetledge S., Jung J.P., Carter R., Sabliov C. (2021). Distribution of polymeric nanoparticles in the eye: Implications in ocular disease therapy. J. Nanobiotech..

[10] Mandal A., Bisht R., Rupenthal I.D., Mitra A.K. (2017). Polymeric micelles for ocular drug delivery: From structural frameworks to recent preclinical studies. J. Control. Release.

[11] Hanafy N.A.N., El-Kemary M., Leporatti S. (2018). Micelles Structure Development as a Strategy to Improve Smart Cancer Therapy. Cancers.

[12] Bose A., Roy Burman D., Sikdar B., Patra P. (2021). Nanomicelles: Types, properties and applications in drug delivery. IET Nanobiotech..

[13] Vaishya R.D., Khurana V., Patel S., Mitra A.K. (2014). Controlled ocular drug delivery with nanomicelles. Wiley Interdiscip. Rev. Nanomed. Nanobiotechnol..

[14] Giuliano E., Paolino D., Fresta M., Cosco D. (2018). Mucosal Applications of Poloxamer 407-Based Hydrogels: An Overview. Pharmaceutics.

[15] Sugumaran A., Ponnusamy C., Kandasamy P., Krishnaswami V., Palanichamy R., Kandasamy R., Lakshmanan M., Natesan S. (2018). Development and evaluation of camptothecin loaded polymer stabilized nanoemulsion: Targeting potential in 4T1-breast tumour xenograft model. Eur. J. Pharm. Sci..

[16] Bodratti A.M., Alexandridis P. (2018). Formulation of Poloxamers for Drug Delivery. J. Funct. Biomater..

[17] Khanal P. (2021). Antimalarial and anticancer properties of artesunate and other artemisinins: Current development. Mon. Chem. Chem. Mon..

[18] Cheong D.H.J., Tan D.W.S., Wong F.W.S., Tran T. (2020). Anti-malarial drug, artemisinin and its derivatives for the treatment of respiratory diseases. Pharmacol. Res..

[19] Wang J., Zhang J., Shi Y., Xu C., Zhang C., Wong Y.K., Lee Y.M., Krishna S., He Y., Lim T.K. (2017). Mechanistic Investigation of the Specific Anticancer Property of Artemisinin and Its Combination with Aminolevulinic Acid for Enhanced Anticolorectal Cancer Activity. ACS Cent. Sci..

[20] Lu B.-W., Xie L.-K. (2019). Potential applications of artemisinins in ocular diseases. Int. J. Ophthalmol..

[21] Chong C.-M., Zheng W. (2016). Artemisinin protects human retinal pigment epithelial cells from hydrogen peroxide-induced oxidative damage through activation of ERK/CREB signaling. Redox Biol..

[22] Ponnusamy C., Sugumaran A., Krishnaswami V., Kandasamy R., Natesan S. (2019). Design and development of artemisinin and dexamethasone loaded topical nanodispersion for the effective treatment of age-related macular degeneration. IET Nanobiotech..

[23] Li H., Hu D., Liang F., Huang X., Zhu Q. (2020). Influence factors on the critical micelle concentration determination using pyrene as a probe and a simple method of preparing samples. R. Soc. Open Sci..

[24] Mohr A., Talbiersky P., Korth H.-G., Sustmann R., Boese R., Bläser D., Rehage H. (2007). A new pyrene-based fluorescent probe for the determination of critical micelle concentrations. J. Phys. Chem. B.

[25] Subramanian N., Ray S., Ghosal S.K., Bhadra R., Moulik S.P. (2004). Formulation design of self-microemulsifying drug delivery systems for improved oral bioavailability of celecoxib. Biol. Pharm. Bull..

[26] Elnaggar Y.S.R., El-Massik M.A., Abdallah O.Y. (2009). Self-nanoemulsifying drug delivery systems of tamoxifen citrate: Design and optimization. Int. J. Pharm..

[27] Ponnusamy C., Krishnaswami V., Sugumaran A., Natesan S. (2014). Simultaneous estimation of artemisinin and dexamethasone in nanodispersions and assessment of Ex-vivo corneal transport study by RP-HPLC. Curr. Pharm. Anal..

[28] Amin K., Dannenfelser R.-M. (2006). In vitro hemolysis: Guidance for the pharmaceutical scientist. J. Pharm. Sci..

[29] Natesan S., Sugumaran A., Ponnusamy C., Jeevanesan V., Girija G., Palanichamy R. (2014). Development and evaluation of magnetic microemulsion: Tool for targeted delivery of camptothecin to BALB/c mice-bearing breast cancer. J. Drug Target..

[30] Subramanian N., Abimanyu S., Vinoth J., Sekar P.C. (2010). Biodegradable Chitosan Magnetic Nanoparticle Carriers for Sub-Cellular Targeting Delivery of Artesunate for Efficient Treatment of Breast Cancer. AIP Conf. Proc..

[31] Danafar H., Jaberizadeh H., Andalib S. (2018). In vitro and in vivo delivery of gliclazide loaded mPEG-PCL micelles and its kinetic release and solubility study. Artif. Cells Nanomed. Biotechnol..

[32] Begum G., Leigh T., Courtie E., Moakes R., Butt G., Ahmed Z., Rauz S., Logan A., Blanch R.J. (2020). Rapid assessment of ocular drug delivery in a novel ex vivo corneal model. Sci. Rep..

[33] Li X., Pan W., Ju C., Liu Z., Pan H., Zhang H., Nie S. (2009). Evaluation of Pharmasolve corneal permeability enhancement and its irritation on rabbit eyes. Drug Deliv..

[34] Pescina S., Govoni P., Potenza A., Padula C., Santi P., Nicoli S. (2014). Development of a Convenient ex vivo Model for the Study of the Transcorneal Permeation of Drugs: Histological and Permeability Evaluation. J. Pharm. Sci..

[35] Velpandian T., Bankoti R., Humayun S., Ravi A.K., Kumari S.S., Biswas N.R. (2006). Comparative evaluation of possible ocular photochemical toxicity of fluoroquinolones meant for ocular use in experimental models. Indian J. Exp. Biol..

[36] Ansari M., Haneef M., Murtaza G., Dyspersja S. (2010). Solid Dispersions of Artemisinin in Polyvinyl Pyrrolidone and Polyethylene Glycol. Adv. Clin. Experimetal Med..

[37] Garg A.K., Sachdeva R.K., Kapoor G. (2013). Comparison of crystalline and amorphous carriers to improve the dissolution profile of water insoluble drug itraconazole. Int. J. Pharm. Bio. Sci..

[38] Vyas V., Sancheti P., Karekar P., Shah M., Pore Y. (2009). Physicochemical characterization of solid dispersion systems of tadalafil with poloxamer 407. Acta Pharm..

[39] Zhang Y., Lam Y.M. (2007). Controlled synthesis and association behavior of graft Pluronic in aqueous solutions. J. Colloid Interface Sci..

[40] Saluja H., Mehanna A., Panicucci R., Atef E. (2016). Hydrogen Bonding: Between Strengthening the Crystal Packing and Improving Solubility of Three Haloperidol Derivatives. Molecules.

[41] Li G., Zhong M., Zhou Z., Zhong Y., Ding P., Huang Y. (2011). Formulation optimization of chelerythrine loaded O-carboxymethylchitosan microspheres using response surface methodology. Int. J. Biol. Macromol..

[42] Singh B., Chakkal S.K., Ahuja N. (2006). Formulation and optimization of controlled release mucoadhesive tablets of atenolol using response surface methodology. AAPS PharmSciTech.

[43] El-Kamel A.H. (2002). In vitro and in vivo evaluation of Pluronic F127-based ocular delivery system for timolol maleate. Int. J. Pharm..

[44] Kim H., Csaky K.G. (2010). Nanoparticle-integrin antagonist C16Y peptide treatment of choroidal neovascularization in rats. J. Control. Release.

[45] Paradkar A., Ambike A., Mahadik K. (2004). Characterization of curcumin-PVP solid dispersion obtained by spray drying. Int. J. Pharm..

[46] Ahuja M., Dhake A.S., Sharma S.K., Majumdar D.K. (2011). Diclofenac-loaded Eudragit S100 nanosuspension for ophthalmic delivery. J. Microencapsul..

[47] Majumdar S., Srirangam R. (2009). Solubility, stability, physicochemical characteristics and in vitro ocular tissue permeability of hesperidin: A natural bioflavonoid. Pharm. Res..

[48] Toropainen E., Ranta V.P., Talvitie A., Suhonen P., Urtti A. (2001). Culture model of human corneal epithelium for prediction of ocular drug absorption. Invest. Ophthalmol. Vis. Sci..

[49] Naguib S.S., Hathout R.M., Mansour S. (2017). Optimizing novel penetration enhancing hybridized vesicles for augmenting the in-vivo effect of an anti-glaucoma drug. Drug Deliv..

